# Formulation of Fiber-Enriched Crackers with Oleaster Powder: Effect on Functional, Textural, and Sensory Attributes

**DOI:** 10.1007/s11130-025-01323-w

**Published:** 2025-02-28

**Authors:** Beyzanur Düşkün, Gozde Kutlu, Perihan Kübra Akman, Hatice Bekiroğlu, Fatih Tornuk

**Affiliations:** 1 Present Address: Department of Food Engineering, Faculty of Chemical and Metallurgical Engineering, Yildiz Technical University, Davutpasa Campus, Istanbul, Türkiye; 2 Faculty of Fine Arts, Design and Architecture, Department of Gastronomy and Culinary Arts, Ankara Medipol University, Ankara, Türkiye; 3 Food Engineering Department, Faculty of Agriculture, Sirnak University, 73300 Sirnak, Türkiye; 4 Faculty of Health Sciences, Department of Nutrition and Dietetics, Sivas Cumhuriyet University, 58140 Sivas, Türkiye

**Keywords:** Oleaster, Crackers, Dietary fiber rich, Glycemic index, Product formulation

## Abstract

The objective of this study was to produce fiber-enriched crackers by substitution of corn flour and whole wheat flour with oleaster powder (OP) at levels of 0% (C-0), 10% (C-10), 20% (C-20), 30% (C-30), and 50% (C-50) and to determine their sensory, textural, color, and bioactive properties, as well as revealing their dietary fiber and glycemic index values. The results showed that the L* values of the samples were found to range from 62.39 (C-30) to 67.31 (C-0), a* values from 4.43 (C-10) to 8.30 (C-50), and b* values from 21.32 (C-10) to 27.40 (C-50). The hardness and fracturability of the samples decreased with increasing OP concentration, with hardness values ranging from 77.45 to 363.35 N and fracturabilitiy values ranging from 23.30 to 33.90 mm. The total phenolic content (TPC) of the cracker samples ranged from 7.39 to 15.06 mg GAE/100 g, while DPPH radical scavenging activity, CUPRAC antioxidant activity, and FRAP assay values increased with the elevating OP concentration, spanning 4.76 to 93.09 mg TE/100 g, 191.03 to 557.4 mg TE/100 g, and 35.70 to 129.90 mg TE/100 g, respectively. The total dietary fiber content also increased in parallel with the OP level (from 6.83 to 15.15%). The Hydrolysis index values ranged from 109.00 to 74.49%. Although all cracker samples were classified as high glycemic index foods, OP incorporation also decreased the glycemic values up to ⁓19% with values ranging from 80.60 (C-50) to 99.55 (C-0). In conclusion, the addition of OP significantly improved the nutritional properties of the crackers by increasing their dietary fiber, TPC and antioxidant activities as well as decreasing their glycemic index without affecting the sensory attributes. Achieving a balance between nutritional improvement and desirable sensory attributes remains key for optimizing cracker formulations.

**Supplementary Information:**

The online version contains supplementary material available at 10.1007/s11130-025-01323-w.

## Introduction

With changing living conditions and increasing education levels, dietary preferences of consumers have also evolved. Globally, there is growing interest on developing high-nutrient, functional foods, leading to a rapid expansion of the healthy snack market. Recent studies have focused on enhancing the nutritional value of baked goods by incorporating various functional ingredients. Cookies and crackers, in particular, have gained popularity due to their low production costs, easy accessibility, and long shelf life [1–3].

*Elaeagnus angustifolia* L. (oleaster), a shrub from the Elaeagnaceae family, is characterized by its small, reddish-brown, elliptical fruits measuring 1.5–2 cm in diameter and sharp-tipped, short-stalked leaves. Known commonly as Russian olive or wild olive due to its resemblance to the olive tree, oleaster is found in over 90 species across Asia, Europe, and parts of North America. In Türkiye, its fruits are widely harvested in Central and Eastern Anatolia during September [4–7]. Oleaster fruit is noted for its significant role in sustaining ecosystem functions in arid regions due to its high tolerance to severe drought, soil salinity, and alkalinity [8]. The fruit is rich in flavonoids, terpenoids, glucose, fructose, and various phenolic acids (such as 4-hydroxybenzoic acid, 4-hydroxycinnamic acid, benzoic acid, caffeic acid, ferulic acid, and vanillic acid), as well as tocopherols, carotenoids, vitamin C, vitamin B1, calcium, magnesium, potassium, iron, and manganese [5, 9, 10].

Crackers are popular baked goods known for their ready-to-eat convenience, extended shelf life, low cost, and variety [11, 12]. Typically made from unleavened dough, they are thin, crispy, and low in sugar, fat, and moisture (2–3%) [2, 13]. The main ingredients of crackers include wheat flour, water, fat, salt, and occasionally a small amount of sugar and leavening agents. Emulsifiers, enzymes, acidity regulators, eggs, sweeteners, and other additives are used to enhance dough processing and the final product’s quality [14]. Crackers are usually made with stronger flours than those used for cookies, and their puffiness, defined as the ratio of pre-baking to post-baking thickness, is a critical quality indicator [15, 16]. Crackers hold significant potential for improving nutritional quality through the addition of functional components and can be formulated with phenolic-rich natural materials to enhance their sensory and physical properties [12, 17, 18].

Based on studies examining the substitution effect of incorporating oleaster into various products, including bread [5], cookies [18], ice cream [7, 9], lavash [19], doughnut [20], kefir [21], and crackers [22], it has been found that different parts of oleaster could serve as a valuable source of composite flour. Specifically, the work by Incedayı & Turkmen-Erol [22] explored the incorporation of oleaster into cracker formulations and assessed its impact on sensory attributes and bioactive features through in vitro digestion studies. As far as we know, there is no study evaluating the dietary fiber content and glycemic index values of crackers incorporated with oleaster flour. Therefore, the current research aimed to address this gap by developing and characterizing crackers with varying oleaster flour content (0, 10, 20, 30, and 50%). The primary objective was to investigate the feasibility of using oleaster flour for producing fiber-enriched functional crackers and to assess how different levels of oleaster flour influence the characteristics and quality of the final product. This comprehensive approach not only evaluates the nutritional benefits of oleaster but also examines its practical implications for enhancing the health attributes and consumer acceptance of cracker products.

## Materials and Methods

### Materials

Fresh oleaster fruits were procured from Ziya Organic Agriculture Enterprises Inc., Lüleburgaz, Kirklareli, Türkiye. The cracker formulation included whole wheat flour (13.7% protein, Gelenek Flour brand, Türkiye), corn flour (7% protein, Nar Spice Corporation, İzmir, Türkiye), labneh cheese (5.6% protein, Milkten brand, Türkiye), yogurt (3.8% protein, Eker Dairy products, Bursa, Türkiye), extra virgin olive oil (Komili company, Ayvalik, Türkiye), and a spice mixture (40% rock salt, 20% oregano, 20% paprika, 20% black pepper), all of which were commercially provided from a local market in Istanbul, Türkiye.

## Preparation of Oleaster Flour

The oleaster shell was peeled by hand and the fleshy parts were separated from the seed using a knife. The pulp was pulverized and then sieved to obtain the ready-to-use oleaster powder (OP) [7].

## Preparation of Crackers

In this study, 5 different cracker formulations were prepared, including four variations with the addition of oleaster powder (OP) and one control sample. The ingredient quantities for these formulations are presented in Table 1. A blend of whole wheat flour and corn flour in a 3:1 ratio was used as the base. Corn flour contributes to the fiber content of crackers, complementing the wheat flour base to create a nutritionally balanced formulation. Through the incorporation of corn flour, the product gains an enhanced fiber profile, supporting the development of a functional, fiber-enriched snack. This combination of flours was optimized through preliminary trials to achieve the best sensory and textural properties, ensuring both nutritional value and consumer acceptance.

**Table 1:** Cracker formulations utilized in the study

Samples	Flour blend (g)	OP (g)	Yogurt (g)	Labneh cheese (g)	Olive oil (mL)	Spice mix (g)
C-0	100	-	45	40	15	1
C-10	90	10	45	40	15	1
C−20	80	20	45	40	15	1
C-30	70	30	45	40	15	1
C-50	50	50	45	40	15	1

*OP* Oleaster powder, *C-0* Crackers without OP, *C-10* Crackers containing 10% OP, *C-20* Crackers containing 20% OP, *C-30* Crackers containing 30% OP, *C-50* Crackers containing 50% OP

The spice mixture consisted of 40% salt, 20% paprika, 20% black pepper, and 20% thyme. To enhance the flavor and texture, prespecified amounts of cracker ingredients including flour and spice mixture, yogurt, labneh cheese, and olive oil were combined. In order to formulate OP supplemented crackers, the flour mixture was replaced by OP at levels of 10, 20, 30, or 50%. The control sample contained no OP, and the cracker samples were coded as follows: C-0 (control), C-10 (10% OP), C-20 (20% OP), C-30 (30% OP), and C-50 (50% OP). The cracker production was carried out at the Pilot Food Production Plant of the Food Engineering Department at Yıldız Technical University. For this aim, the dry ingredients (whole wheat flour, corn flour, OP, and spice mixture) were mixed at high speed for 1 min using a kitchen mixer (KitchenAid, USA) to ensure homogeneity. Subsequently, the remaining ingredients were added, and the dough was mixed at medium speed for an additional 3 min. The dough was then held at room temperature for 30 min. To prevent sticking, the dough was placed between two sheets of parchment paper and rolled out to a thickness of approximately 0.3 cm using a household pasta machine. The dough was then cut into equal squares (4 cm x 4 cm) with a rotary cutter. The cracker dough was baked in an oven (Fimak Estone, Türkiye) at 150 °C for 15 min. After baking, the samples were cooled at ambient temperature before further analyses.

 This section is given in Supplementary Material (SM).

## Results and Discussion

### Color Characteristics of Cracker Samples

The L*, a*, and b* values of the cracker samples, as presented in Table 2, showed statistically significant differences (*p* < 0.05). The L* values, representing lightness, decreased with increasing OP levels. Specifically, the C-30 sample had the lowest L* value at 62.39 ± 0.55, while the highest L* value of 67.31 ± 0.78 was recorded for the C-0 sample. There was no significant difference (*p* > 0.05) between the L* values of the C-20 and C-50 samples. Previous studies suggested that the reduction in L* values with increasing OP content in baked goods could be attributed to Maillard reactions, which cause the product to darken [23], the formation of brown pigments in OP [7], and the substitution of wheat flour with fiber-rich flours containing sugars, which can enhance non-enzymatic browning and contribute to the observed effect [24]. This aligns with the findings of Yavuz et al. [5], who noted that higher OP levels resulted in darker bread. Similar trends were observed in OP-substituted ice cream [7, 9], and doughnuts [20], where higher OP content led to darker products.

**Table 2:** Color and texture attributes of C-0, C-10, C-20, C-30, and C-50 crackers

Samples	Color properties				Texture properties	
	L*	a*	b*	∆E*	Hardness (*N*)	Fracturability (mm)
C-0	67.31 ± 0.78^c^	5.28 ± 0.15^b^	24.43 ± 0.87^bc^	-	363.35 ± 6.20^d^	33.90 ± 0.20^b^
C-10	66.66 ± 0.44^c^	4.43 ± 0.37^a^	21.32 ± 0.55^a^	3.28	289.36 ± 21.30^c^	33.73 ± 0.50^b^
C-20	64.17 ± 0.68^b^	7.22 ± 0.51^d^	25.08 ± 0.70^c^	3.75	246.98 ± 8.60^b^	33.17 ± 1.20^b^
C-30	62.39 ± 0.55^a^	6.07 ± 0.24^c^	23.61 ± 0.52^b^	5.05	239.17 ± 20.60^b^	32.43 ± 0.50^b^
C-50	64.93 ± 0.76^b^	8.30 ± 0.61^e^	27.40 ± 0.65^d^	4.90	77.45 ± 10.08^a^	23.30 ± 1.08^a^

*OP* Oleaster powder, *C-0* Crackers without OP, *C-10* Crackers containing 10% OP, *C-20* Crackers containing 20% OP, *C-30* Crackers containing 30% OP, *C-50* Crackers containing 50% OP

The results revealed that the C-50 and C-10 samples exhibited the highest and the lowest a* values, respectively. Increasing OP concentration resulted in significantly higher a* values (*p* < 0.05). Similarly, raising the proportion of OP in food formulations resulted in higher a* values [7, 21], indicating that the color of the enriched crackers shifted towards red.

Regarding the b* values, a similar trend with the a* values was observed. As seen in Table 2, C-50 and C-10 samples had the highest and the lowest b* values. Statistically significant differences were observed among all samples (*p* < 0.05). This trend was consistent with studies on OP in ice cream [7], kefir [21], and bread [5], where increasing OP content led to higher b* values.

Color difference (ΔE*) analysis revealed that the C-30 sample exhibited the greatest color deviation. As expected, ΔE* values of the crackers were proportional to their OP concentrations. In all samples, as the ΔE* values were > 3, the color differences were perceptible by human eye [24].

Crust color in bakery products is strongly influenced by Maillard and caramelization reactions, as well as ingredients used in the formulation. Key factors such as baking temperature and residual sugar levels play a significant role in determining the final color [5]. Similarly, as free amino acid and reducing sugar levels increase, the Maillard reaction intensifies, leading to a reduction in the L* value. Additionally, lower pH levels accelerate this reaction by boosting the production of reducing sugars and promoting furfural formation [25]. Additionally, Gül et al. [21] noted that insoluble particles, which function as light-scattering elements, and the color of raw materials in the formulation also influence the final color properties of products.

### Textural Properties of Cracker Samples

Table 2 presents the textural properties of the cracker samples as affected by OP concentration. Hardness, defined as a product’s resistance to deformation or its firmness, showed statistically significant differences (*p* < 0.05) between the cracker formulations. According to the findings, the control sample (C-0) exhibited the highest hardness (363.35 ± 6.2 N) while it decreased depending on the OP concentration. All the differences in hardness values between the samples except for the difference of C-20 and C-30 were significant. The decrease in hardness with the increasing OP concentration may be due to the decreasing gluten content, which led to a weaker and more crumbly structure [25]. It may be also caused by higher water retention during baking and the inhibitory effects of OP’s phenolic compounds on amylase activity [2], leading to a softer, less firm texture. Another phenomenon may be that the inclusion of dietary fibers in cracker formulations reduced the hardness of the final products [26]. In general, reduced hardness for crackers is not desired by consumers who seek crisp crackers with a lighter texture.

Fracturability, which refers to the distance at which a product breaks, reflects the crispness of crackers. Crackers with shorter breaking distances tend to have higher fracturability [27]. As shown in Table 2, fracturability values followed a similar trend to hardness. The control sample (C-0) had the highest fracturability (33.90 ± 0.20 mm), followed by C-10 (33.73 ± 0.50 mm), C-20 (33.17 ± 1.20 mm), C-30 (32.43 ± 0.50 mm), and C-50 (23.30 ± 1.08 mm). However, no significant difference between the fracturability values was observed (p > 0.05), except for the C-50 formulation which had the lowest values for both hardness and fracturability. Decrease in fracturability suggested that crackers were less brittle and could break into fewer pieces, potentially compromising their desired crispness and crunchiness [28]. This observation aligns with previous research by Howard et al. [22], where crackers made with peanut flour were similarly found to be softer compared to the commercial reference samples. The reduced water availability for gluten development in OP-containing crackers may have hindered gluten matrix formation, resulting in weaker dough and reduced structural integrity [25].

## Bioactive Properties of Cracker Samples

### TPC

In the present study, TPC of the cracker samples ranged from 7.39 ± 0.13 to 15.06 ± 0.31 mg GAE/100 g (Table 3). The C-0 sample exhibited the lowest TPC, while a statistically significant increase (*p* < 0.05) in TPC was observed with the increasing OP content, demonstrating a positive correlation between OP concentration and the phenolic content of the enriched crackers.

**Table 3:** Bioactive properties of C-0, C-10, C-20, C-30, and C-50 crackers

Samples	TPC (mg GAE/ 100 g)	Total antioxidant capacity (mg TE/100 g)			
		DPPH activity	CUPRAC activity	FRAP activity	
C-0	7.39 ± 0.13^a^	4.76 ± 1.25^a^	191.03 ± 0.80^a^	35.70 ± 1.40^a^	
C-10	8.99 ± 0.38^b^	17.75 ± 3.03^b^	274.10 ± 8.90^b^	41.80 ± 0.40^b^	
C-20	11.33 ± 0.14^c^	37.23 ± 1.52^c^	307.20 ± 15.40^c^	71.20 ± 0.50^c^	
C-30	12.01 ± 0.21^c^	42.14 ± 0.95^c^	365.10 ± 4.04^d^	78.20 ± 0.80^d^	
C-50	15.06 ± 0.31^d^	93.09 ± 2.12^d^	557.40 ± 4.80^e^	129.90 ± 2.80^e^	

*OP* Oleaster powder, *C-0* Crackers without OP, *C-10* Crackers containing 10% OP, *C-20* Crackers containing 20% OP, *C-30* Crackers containing 30% OP, *C-50* Crackers containing 50% OP, *TE* Trolox equivalent, *GAE* Gallic acid equivalent

These findings align with previous studies, such as those by Gül et al. [21], Incedayı & Turkmen-Erol [22], and Süren et al. [7], who reported higher TPC levels in enriched products with OP. OP is known for its high phenolic content, primarily consisting of gallic acid, catechin, and their derivatives [29]. Enrichment of foods with phenolic compounds originating from OP could also contribute to their antioxidant properties of the crackers, as phenolics are well-documented for their health-promoting potential due to their antioxidative and radical-scavenging activities.

Moreover, Incedayı & Turkmen-Erol [22] demonstrated a significant increase (*p* < 0.05) in TPC with OP supplementation in crackers, with the highest increase observed at 15% OP addition. This suggested that the phenolic content of the crackers could be substantially improved by fortifying them with OP, leading to a notable enhancement in their antioxidant capacity. The increase in TPC with OP addition could be attributed to the high phenolic concentration in OP, which enhanced the nutritional value of the crackers.

### Antioxidant Activities

Table 3 presents the antioxidant activity results for cracker samples evaluated through DPPH, CUPRAC, and FRAP assays. In general, antioxidant activities were well correlated with the OP supplementation of the crackers, which demonstrates its antioxidant potential. DPPH radical scavenging activity (mg TE/100 g) increased proportionally with OP concentration (Table 3). These results demonstrated the strong correlation between TPC and antioxidant capacity. Furthermore, CUPRAC values ranged from 191.03 ± 0.8 mg TE/100 g in C-0 to 557.4 ± 4.8 mg TE/100 g in C-50 (Table 3). The consistent increase in antioxidant activity across all assays underscores the significant role of phenolic compounds in enhancing the overall antioxidant capacity [14]. The FRAP results were showed 35.70 ± 1.40 mg TE/100 g in C-0, 41.80 ± 0.40 mg TE/100 g in C-10, 71.20 ± 0.5 mg TE/100 g in C-20, 78.20 ± 0.80 mg TE/100 g in C-30, and 129.90 ± 2.80 mg TE/100 g in C-50 (Table 3).

Farzaei et al. [30] reported that flavonoid glycosides such as isorhamnetin-3-O-β-D-galactopyranoside-4’-O-β-D-glucopyranoside, quercetin 3-O-β-D-galactopyranoside-4’-O-β-D-glucopyranoside, and quercetin 3,4’-O-β-D-diglucoside found in oleaster fruits exhibit strong concentration-dependent antioxidant activity, effectively scavenging DPPH and ABTS^+^ radicals. These findings further support the results of this study. In line with previous studies that demonstrated significant improvements in the antioxidant properties of foods enriched with OP—such as ice cream [5, 7], cookies [18], and kefir [21]—this research reinforces the potential of OP as a valuable bioactive ingredient [31].

### Dietary Fiber Content of Cracker Samples

The results indicated that the C-0 and C-50 samples had the lowest (6.83 ± 0.08%) and the highest total dietary fiber (TDF) contents (15.15 ± 0.52%), respectively (Table 4), resulting in an increase of approximately 121.82% in TDF content. A significant correlation was found between the level of OP and the TDF content of the crackers (*p* < 0.05). However, no statistically significant difference in TDF content was observed between the C-0 and C-10 samples (*p* > 0.05). These findings were consistent with the fact that OP contained considerable amounts of dietary fiber [5, 6]. The dietary fibers present in the control samples likely originated from the whole wheat flour and corn flour included in the formulation. A 40-gram serving of the C-0 sample contains 2.7% TDF, which accounts for approximately 10.93% of the daily recommended intake of dietary fiber. However, a 40-gram serving of the C-50 sample contains 6.06% TDF, providing around 24.24% of the daily recommended dietary fiber intake.

**Table 4:** Total dietary fiber, HI and GI values of C-0, C-10, C-20, C-30, and C-50 crackers

Samples	Total dietary fiber (%)	HI (%)	GI (%)
Bread	-	96.63 ± 0.92^c^	92.76 ± 0.40^c^
C-0	6.83 ± 0.08^d^	109.00 ± 2.17^a^	99.55 ± 1.34^a^
C-10	7.49 ± 0.38^d^	103.10 ± 1.15^b^	96.31 ± 0.80^b^
C-20	10.79 ± 0.87^c^	97.42 ± 1.06^c^	93.19 ± 0.50^c^
C-30	12.48 ± 0.58^b^	80.62 ± 1.05^d^	83.97 ± 0.50^d^
C-50	15.15 ± 0.52^a^	74.49 ± 0.60^e^	80.60 ± 0.94^e^

*OP* Oleaster powder, *C-0* Crackers without OP, *C-10* Crackers containing 10% OP, *C-20* Crackers containing 20% OP, *C-30* Crackers containing 30% O, *C-50* Crackers containing 50% OP, *HI* Hydrolysis index, *GI* Glycemic index

According to the World Health Organization, adults are recommended to consume at least 25 g of dietary fiber per day, with products containing at least 3% dietary fiber labeled as a “source of fiber” and those with over 6% as “high in fiber” [32]. Based on this classification, all samples, including the C-0 samples, can be categorized as high-fiber crackers due to their TDF values exceeding 6%. The general population often fails to meet the recommended fiber intake, which can contribute to increasing obesity rates [33]. Similarly, substituting wheat flour with OP resulted in higher TDF content in cookies compared to the reference samples [23].

### Impact of OP-enriched Crackers on Hydrolysis Index (HI) and Glycemic Response

According to the results, the HI values were 96.63 ± 0.92% for bread, 109.00 ± 2.17% for C-0, 103.10 ± 1.15% for C-10, 97.42 ± 1.06% for C-20, 80.62 ± 1.05% for C-30, and 74.49 ± 0.60% for C-50 (Table 4). While no statistically significant difference (*p* > 0.05) was found between bread and C-10, the differences among the other samples were significant (*p* < 0.05). The inclusion of OP, particularly at higher concentrations (C-30 and C-50), effectively reduced HI values, suggesting that OP slowed down carbohydrate digestion and could potentially lower the glycemic index of the crackers. This functional role of OP was evident in the significant HI reduction observed with higher OP levels, demonstrating the potential for OP to moderate glycemic response.

In this study, the GI values ranged from 80.60 ± 0.94% (C-50) to 99.55 ± 1.34% (C-0) (Table 4), placing all samples within the high GI category. The rankings, from lowest to highest, were as follows: C-50 < C-30 < Bread < C-20 < C-10 < C-0. Notably, the difference between C-20 and bread was not statistically significant (*p* > 0.05), while all other differences were significant (*p* < 0.05). The addition of 50% OP (C-50) reduced the GI value by approximately 19.04% compared to the control crackers, demonstrating that OP-enriched crackers produced a lower glycemic response. This implies that these crackers may lead to a more gradual increase in postprandial blood glucose levels, making them more suitable for individuals seeking to manage their blood sugar levels.

Lower GI values by OP incorporation can be also attributed to the inhibitory effects of phenolic compounds on carbohydrate-digesting enzymes, as previously described by Erol et al. (2024) [34]. OP, being rich in phenolic compounds, likely contributed to the reduced GI values in these samples. According to Erol et al. [34], foods are classified into three categories based on GI values: low (< 55), medium (55–69), and high (> 70). Despite the reductions in GI, all cracker samples, including those enriched with OP, still fall within the high GI category (80.6 ± 0.94 to 99.55 ± 0.92).

In conclusion, OP-enriched crackers, particularly those with 30% and 50% OP, exhibit lower HI and GI values compared to control samples. While all the samples remain within the high GI range, the significant reductions in both HI and GI with increasing OP levels underscore the potential of OP to improve the glycemic profile of crackers. These findings were consistent with previous studies, such as Heidari et al. [35], who reported that OP supplementation in sugar-free biscuits reduced the GI.

### Sensory Analysis

The color values of the cracker samples ranged from 5.80 ± 0.70 (C-50) to 6.80 ± 0.40 (C-0 and C-10), while taste-aroma scores varied between 6.70 ± 0.50 (C-50) and 6.90 ± 0.30 (C-10 and C-20). Odor values also ranged from 6.70 ± 0.50 (C-50) to 6.90 ± 0.30 (C-10 and C-20), with taste-flavor scores ranging from 4.60 ± 1.20 (C-50) to 6.20 ± 0.70 (C-20) (Table 5). Crispness scores were recorded as 3.40 ± 0.90 (C-50) to 6.90 ± 0.30 (C-0), and overall acceptability ranged from 4.70 ± 0.80 (C-50) to 6.50 ± 0.50 (C-0) (Table 5).

**Table 5:** Sensory attributes of C-0, C-10, C-20, C-30, and C-50 crackers

Samples	Color	Odor	Taste & aroma	Crispiness	General acceptability
C-0	6.80 ± 0.40^b^	6.80 ± 0.40^a^	5.90 ± 0.70^b^	6.90 ± 0.30^b^	6.50 ± 0.50^b^
C-10	6.80 ± 0.40^b^	6.90 ± 0.30^a^	6.00 ± 0.70^b^	6.20 ± 0.60^b^	6.30 ± 0.60^b^
C-20	6.50 ± 0.50^ab^	6.90 ± 0.30^a^	6.20 ± 0.70^b^	6.70 ± 0.40^b^	6.30 ± 0.90^b^
C-30	6.50 ± 0.50^ab^	6.80 ± 0.40^a^	5.70 ± 1.10^ab^	6.20 ± 0.40^b^	5.90 ± 0.90^b^
C-50	5.80 ± 0.70^a^	6.70 ± 0.50^a^	4.60 ± 1.20^a^	3.40 ± 0.90^a^	4.70 ± 0.80^a^

*OP* Oleaster powder, *C-0* Crackers without OP, *C-10* Crackers containing 10% OP, *C-20* Crackers containing 20% OP, *C-30* Crackers containing 30% O, *C-50* Crackers containing 50% OP

A significant relationship was found between OP content and overall acceptability (*p* < 0.05), with C-0 receiving the highest score and acceptability. The decrease in general acceptability, particularly for C-50, was primarily attributed to changes in taste-aroma and texture, as panelists noticed the reduction in crispness with increasing OP content. This was consistent with the results given by Öztürk et al. [36], who reported that modifications in yogurt with oleaster flour, such as reduced brightness and increased redness and yellowness, resulted in lower sensory scores for appearance. Similarly, the addition of OP significantly impacted the texture of the crackers in this study. However, these findings contrast with those of Incedayı & Türkmen-Erol [22], who observed higher organoleptic scores for crackers containing OF compared to the control sample. This discrepancy may be due to differences in cracker formulation. Consistent with the textural analysis results, increasing OP content led to a reduction in hardness, negatively influencing the sensory evaluation of texture and resulting in lower scores. While these textural changes may have impacted overall sensory perception, balancing improved nutritional benefits with sensory quality remains a key challenge in product development.

## Conclusion

The current study aims to investigate the effects of incorporating oleaster powder (OP) into crackers at ratios of 10, 20, 30, and 50% as a flour substitute, comparing the OP-enriched crackers with control samples without substitutes. Sensory, textural, color, and bioactivity properties of the cracker samples were determined, while changes in dietary fiber and glycemic index values with the addition of OP were also examined. The findings indicated that the incorporation of OP into cracker formulations significantly enhanced their nutritional attributes, particularly in terms of dietary fiber and antioxidant activity, while reducing the glycemic index. A significant positive relationship was observed between the amount of OP incorporated into the crackers and their antioxidant capacity. However, the sensory and textural properties were negatively impacted, with higher OP levels leading to lower overall acceptability. These findings highlighted the potential of OP as a functional ingredient in cracker production but also underscore the need to carefully balance health benefits with sensory appeal. Future research should focus on optimizing formulations to enhance both the nutritional value and consumer acceptance of OP-enriched crackers.

## Supplementary Information

Below is the link to the electronic supplementary material.

ESM 1 (DOCX 29.0 KB)

## Data Availability

No datasets were generated or analysed during the current study.

## Abbreviations

OP: Oleaster powder
TPC: Total phenolic content
C-0: Crackers without oleaster powder
C-10: Crackers containing 10% oleaster powder
C-20: Crackers containing 20% oleaster powder
C-30: Crackers containing 30% oleaster powder
C-50: Crackers containing 50% oleaster powder
