# Supplementary material for: Formulation of Fiber-Enriched Crackers with Oleaster Powder: Effect on Functional, Textural, and Sensory Attributes
Source: Plant Foods Hum Nutr. 2025 Feb 28;80(1):82. doi: 10.1007/s11130-025-01323-w (PMC11870954; doi:10.1007/s11130-025-01323-w)
Supplement: Supplementary file 1 — (DOCX 29.0 KB) [file 11130_2025_1323_MOESM1_ESM.docx]

**Formulation of fiber-enriched crackers with oleaster powder: Effect on functional, textural, sensory, and nutritional attributes**

Beyzanur Düşkün^a^, Gozde Kutlu^b^, Perihan Kübra Akman^c^, Hatice Bekiroğlu^d^, Fatih Tornuk^e,*^

^a^ Yildiz Technical University, Faculty of Chemical and Metallurgical Engineering, Department of Food Engineering. Davutpasa Campus, Istanbul, Türkiye. ✉: beynurdem@gmail.com [
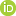
](https://orcid.org) 0000-0003-2259-4893

^b^ Ankara Medipol University, Faculty of Fine Arts, Design and Architecture, Department of Gastronomy and Culinary Arts, Ankara, Türkiye. ✉: gozcelk@gmail.com [
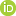
](https://orcid.org) 0000-0001-7111-1726

^c^ Yildiz Technical University, Faculty of Chemical and Metallurgical Engineering, Department of Food Engineering, Davutpasa Campus, Istanbul, Türkiye. ✉: pkcicek@yildiz.edut.tr [
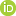
](https://orcid.org) 0000-0002-7837-4681

^d^ Sirnak University, Food Engineering Department, Faculty of Agriculture, 73300, Sirnak, Türkiye. Yildiz Technical University, Faculty of Chemical and Metallurgical Engineering, Department of Food Engineering. Davutpasa Campus, Istanbul, Türkiye. ✉: h.bkroglu@gmail.com [
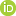
](https://orcid.org) 0000-0003-3328-1550

^e^ Sivas Cumhuriyet University, Faculty of Health Sciences, Department of Nutrition and Dietetics, 58140, Sivas, Türkiye. ✉: ftornuk@cumhuriyet.edu.tr [
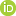
](https://orcid.org) 0000-0002-7313-0207

**Materials and Methods**

**Color measurement**

The color parameters of the crackers were analyzed using a colorimeter (Minolta CR-400, Konica Minolta Sensing, Osaka, Japan). The *L** (lightness: white-black), *a** (red-green), and *b** (yellow-blue) values were measured in triplicate for each sample following established protocols [1]. The total color difference *(∆E*)* was calculated using the following formula:

| $\Delta E^{*}=\sqrt{\left( {\Delta a}^{*} \right)^{2}+\left( {\Delta b}^{*} \right)^{2}+\left( {\Delta L}^{*} \right)^{2}}$ | (1) |
| --- | --- |

**Texture analysis**

The textural properties of the crackers, including hardness and brittleness, were assessed using a texture profile analyzer (SMS, TA-HD Plus, UK) equipped with a triple-point probe. The parameters for the analysis were set as follows: pre-test speed of 1 mm/s, test speed of 3 mm/s, post-test speed of 10 mm/s, a compression distance of 5 mm, and a trigger force of 50 g [1]. The maximum force (N) required to break the cracker samples was recorded.

**Determination of bioactive properties**

**Extraction**
Cracker samples were ground into a fine powder using a coffee-spice grinder (Fakir, Germany). A 10 g portion of the powdered sample was extracted with 40 mL of 80% methanol (Merck) by stirring the mixture for 24 h on a magnetic stirrer. The mixture was then filtered through a 0.45 µm filter to obtain the liquid extracts [2].

**Determination of total phenolic content (TPC)**

The TPC of the cracker extracts was determined using the Folin-Ciocalteu spectrophotometric method. Each reaction tube contained 2.5 mL of 0.2 N Folin reagent (Merck), 2 mL of Na_2_CO_3_ (Sigma Aldrich), and 0.5 mL of the extract. The mixtures were incubated in the dark at room temperature for 30 min. After the incubation, the absorbance was measured at 760 nm using a UV-visible spectrophotometer (Shimadzu UV-1800) [3].

**Determination of DPPH radical scavenging activity**

The DPPH radical scavenging activity was measured using the 1,1-diphenyl-2-picrylhydrazyl (DPPH) method. In test tubes, 0.1 mL of the extract was mixed with 4.9 mL of DPPH solution (Sigma Aldrich). The mixture was incubated in the dark at room temperature for 20 min, after which the absorbance was recorded at 517 nm [3].

**Determination of total antioxidant capacity by CUPRAC method**

The total antioxidant capacity was determined using the CUPRAC method. In test tubes, 1 mL of Cu (II) chloride (Merck), 1 mL of neocuproine (Sigma Aldrich), 1 mL of ammonium acetate buffer (Merck), and 100 µL of the sample were added, followed by 1 mL of distilled water. The mixture was incubated at room temperature for 30 min, and the absorbance was measured at 450 nm [4].

**FRAP method**

FRAP assay was performed by mixing 100 µL of the sample with 900 µL of distilled water. Then, 2 mL of the prepared FRAP reagent was added to each sample. The mixtures were incubated at 37°C for 8 min, and the absorbance was measured at 593 nm [5].

**Total dietary fiber content**

The total dietary fiber content was determined following the AOAC official method (AOAC 985.29) previously applied by McCleary et al. [6]. Ground cracker samples were first defatted by washing three times with 15 mL of chloroform (Merck) and then dried at 105°C for 24 h. Then, 1 g of the dried, defatted sample was mixed with 40 mL of PBS buffer (Sigma Aldrich, Germany) with a pH adjusted to 8.3 ± 0.2 using HCl (Merck, Germany) and 6 M NaOH (Merck, Germany). Subsequently, 50 µL of α-amylase (Merck) was added, and the mixture was incubated at 90°C for 30 min. After cooling to 60°C, 50 µL of protease (Merck, Germany) was added, and the mixture was incubated at 60°C for another 30 min. The pH was then adjusted to 4.0-4.7 using 0.56 M HCl and the addition of 150 µL amyloglucosidase (Merck, Germany). The mixture was incubated at 60°C for 30 min, followed by the addition of 220 mL of 95% ethanol (Merck, Germany). After standing at room temperature for 1 h, the samples were filtered through Gooch crucibles containing pre-weighed celite. The residues were washed with 78% ethanol, 95% ethanol, and acetone (Merck, Germany), then dried at 105°C for 24 h. The dried residues were subsequently used for ash determination and protein analysis using the Kjeldahl method.

**Calculation of glycemic index value**

The glycemic index (GI) of the cracker samples was determined using an *in vitro* method, adapted from Englyst et al. [7], with minor modifications. The preparation of enzymes and solutions followed the protocol described by Yaman et al. [8]. The glycemic index was measured using the Megazyme D-Glucose (GODOP Format) Test Kit. Homogenized samples (1 g) were placed into 50 mL Falcon tubes, and 5 mL of distilled water along with 10 mL of freshly prepared Enzyme Solution 1 (pepsin/guar gum solution) were added. The mixture was vortexed and incubated in a shaking water bath at 37°C for 30 min to allow protein hydrolysis by pepsin. After incubation, the tubes were cooled for 30 min, followed by the addition of 5 mL of 0.5 M sodium acetate solution to adjust the pH to 5.2. Then, 5 mL of Enzyme Solution 2 was added, vortexed, and the final volume was brought to 50 mL with distilled water. The tubes were incubated at 37°C in a water bath, and aliquots of 0.5 mL were taken at 30, 60, 90, and 120 min. The samples were transferred to 10 mL glass tubes, adjusted to a 5 mL volume with 66% (v/v) aqueous ethanol, and vortexed. A 0.1 mL aliquot of each sample was transferred to another glass tube, to which 3 mL of GOPOD (glucose oxidase peroxidase) reagent was added. The tubes were then incubated at 50°C for 15 min. Absorbance was measured at 510 nm using a spectrophotometer, and the glucose content was determined. The estimated GI was calculated from the hydrolysis index (HI), which was derived by dividing the area under the hydrolysis curve of the test sample by the area for white bread [9]. The estimated GI was then computed using an empirical formula.

$GI=39.71+0.549 HI$ (2)

**Sensory analysis**

The sensory evaluation of the cracker samples was conducted by a panel of 13 individuals selected from the students and faculty of the Department of Food Engineering at Yıldız Technical University. Sensory assessments were carried out in a well-illuminated, neutral environment to minimize external influences. The crackers were presented randomly, coded, and served on plastic plates. The panelists were asked to rate the crackers on five attributes: color, aroma, taste-flavor, crispness, and overall acceptability. Each attribute was scored using a 7-point hedonic scale, where 1 corresponded to "disliked very much" and 7 to "liked very much".

**Statistical analysis**

All experiments were performed in triplicate with three replicates for each measurement. Data were recorded in Microsoft Excel, where the means and standard deviations were calculated. Statistical analyses were performed using analysis of variance (ANOVA) with the SPSS software (IBM SPSS Statistics 26 for Windows). Significant differences between means were determined by Tukey's multiple comparison test at a 95% confidence level.

**References**

1. Sahan Y, Dundar AN, Aydin E, Kilci A, Dulger D, Kaplan FB, Gocmen D, Celik G (2013) Characteristics of cookies supplemented with oleaster (*Elaeagnus angustifolia* L.) Flour. I physicochemical, sensorial and textural properties. J Agric Sci 5:160.

2. Kumar I, Sharma RK (2021) Antioxidant properties in methanol extract of kernels of commonly marketed Indian fruits. Int J Fruit Sci, 21:334-343.

3. Aslam HKW, Raheem MIU, Ramzan R, Shakeel A, Shoaib M, Sakandar HA (2014) Utilization of mango waste material (peel, kernel) to enhance dietary fiber content and antioxidant properties of biscuit. J Glob Innov Agric Soc Sci 2:76-81.

4. Cansev Z, Sabuncu M, Gülkun G, Ateş M, Cansev A, Şahan Y (2024) Enginar yan ürünleri ile zenginleştirilmiş bisküvilerin fizikokimyasal ve fonksiyonel özelliklerinin değerlendirilmesi. Gıda Yem Bil Teknol Derg 1-11.

5. Karkar B, Şahin S (2022) Determination of phenolic compounds profiles and antioxidant properties of oleaster (*Elaeagnus angustifolia* L.) grown in Turkey. Eur Food Res Technol 248:219-241.

6. McCleary BV, DeVries JW, Rader JI, Cohen G, Prosky L, Mugford DC, Champ M, Okuma, K (2012) Determination of insoluble, soluble, and total dietary fiber (CODEX definition) by enzymatic-gravimetric method and liquid chromatography: collaborative study. J AOAC Int 95:824-844.

7. Englyst KN, Vinoy S, Englyst HN, Lang V (2003) Glycaemic index of cereal products explained by their content of rapidly and slowly available glucose. Br J Nutr 89:329-339.

8. Yaman M, Sargın HS, Mızrak ÖF (2019) Free sugar content, in vitro starch digestibility and predicted glycemic index of ready-to-eat breakfast cereals commonly consumed in Turkey: An evaluation of nutritional quality. Int J Biol Macromol 135:1082-1087.

9. Bekiroglu H, Komurlu E, Cebi N, Sagdic O (2022) The effect of using resistant starch on glycemic index and rheological properties of milky pudding: low-glycemic index milky pudding. Lat Am Appl Res 52:1-6.
